# Planning and understanding the intensive care network in the State of Rio de Janeiro (RJ), Brazil: a complex societal problem

**DOI:** 10.5935/0103-507X.20180053

**Published:** 2018

**Authors:** Rosane Sonia Goldwasser, Maria Stella de Castro Lobo, Edilson Fernandes de Arruda, Simone Audrey Angelo, Eliana Claudia de Othero Ribeiro, José Roberto Lapa e Silva

**Affiliations:** 1 Hospital Universitário Clementino Fraga Filho, Universidade Federal do Rio de Janeiro - Rio de Janeiro (RJ), Brasil.; 2 Instituto Alberto Luiz Coimbra, Escola de Graduação e Pesquisa em Engenharia, Universidade Federal do Rio de Janeiro - Rio de Janeiro (RJ), Brasil.; 3 Núcleo de Tecnologia Educacional para a Saúde, Universidade Federal do Rio de Janeiro - Rio de Janeiro (RJ), Brasil.; 4 Programa de Pós-Graduação, Faculdade de Medicina, Universidade Federal do Rio de Janeiro - Rio de Janeiro (RJ), Brasil.

**Keywords:** Intensive care unit organization & administration, Management, Health policy, planning and management, Hospital bed capacity, Health services accessibility, Unified health system, Qualitative research

## Abstract

**Objectives:**

To determine the optimal number of adult intensive care unit beds to reduce patient's queue waiting time and to propose policy strategies.

**Methods:**

Multimethodological approach: (a) quantitative time series and queueing theory were used to predict the demand and estimate intensive care unit beds in different scenarios; (b) qualitative focus group and content analysis were used to explore physicians' attitudes and provide insights into their behaviors and belief-driven healthcare delivery changes.

**Results:**

A total of 33,101 requests for 268 regulated intensive care unit beds in one year resulted in 25% admissions, 55% queue abandonment and 20% deaths. Maintaining current intensive care unit arrival and exit rates, there would need 628 beds to ensure a maximum wait time of six hours. A reduction of the current abandonment rates due to clinical improvement or the average intensive care unit length of stay would decrease the number of beds to 471 and 366, respectively. If both were reduced, the number would reach 275 beds. The interviews generated 3 main themes: (1) the doctor's conflict: fair, legal, ethical and shared priorities in the decision-making process; (2) a failure of access: invisible queues and a lack of infrastructure; and (3) societal drama: deterioration of public policies and health care networks.

**Conclusion:**

The queue should be treated as a complex societal problem with a multifactorial origin requiring integrated solutions. Improving intensive care unit protocols and reengineering the general wards may decrease the length of stay. It is essential to redefine and consolidate the regulatory centers to organize the queue and provide available resources in a timely manner, by using priority criteria, working with stakeholders to guarantee clinical governance and network organization.

## INTRODUCTION

Intensive care units (ICU) are key components in the life-saving and life-sustaining management of patients at-risk for imminent death. As critically ill patients need early interventions to improve their outcomes, delayed ICU bed availability results in a negative impact on clinical outcomes and higher mortality rates.^(1)^ Due to the high degree of complexity, the ICU is an expensive resource within the hospital, accounting for at least 20% of hospital admission costs.^(2)^

**Figure 1:**
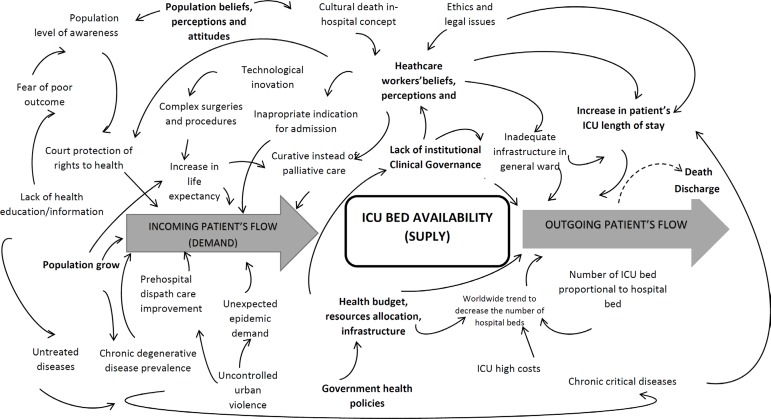
A diagrammatic representation of the complex human situation of intensive care unit demand/supply chain, capturing the key elements of the problem, while considering a variety of stakeholder perspectives. ICU - intensive care unit.

Compared to automated industrial plants, an ICU has a much less predictable planning system; in addition to timing, costs and institutional responsibilities, there is a clear gap between the supply and demand for ICU beds.^(3)^ When the number of new admission requests exceeds the available beds, some patients will be rejected, and a waiting queue is formed. In this sense, we face a real-world stock and flow problem, in which demand (the inflow of patients) has a multitude of interrelated causes (e.g., aging, population perception and epidemiologic demand) and the definition of the optimal stock (the number of beds) and outflow also depends on the connection between technical and human-driven processes, such as regulation and clinical governance. Furthermore, there is a historical tendency towards a decline in the number of total hospital beds, obstructing the outflow from ICU settings.^(4)^ Additionally, the inadequate infrastructure, absence of protocols and lack of trained human resources may contribute to a patient's prolonged length of stay (LOS) and result in flow bottlenecks. Factors that influence demand and supply for the ICU are nonlinear, as demonstrated in figure 1.

Despite the complexity of the issue, an estimation of the number of required ICU hospital beds remains mainly based on the average population as a proxy for epidemiological demand, without considering the perception of the stakeholders.^(5)^

To cope with the access limitations, a few strategies have been created by the Urgency & Emergency Network of the Brazilian Unified Health System (*Sistema* Único *de Saúde* - SUS). These strategies are mainly based on regulatory policies, such as a regulatory center, the *Central Estadual de Regulação* (CER). The *Central Estadual de Regulação* has the goal of improving citizens' orderly admission to high- and medium-complexity services and procedures. It manages existing health demands and available resources to offer the best care within an opportune time frame.^(6)^ Regulation of ICU beds is based on technical priority criteria following hierarchical protocols that were built based on consensus by medical specialty societies, giving priority to patients with more severe illnesses.^(7)^

Given the existence of its many interconnected elements, which are not necessarily connected by linear relationships, the critical care network should be considered a complex societal problem. The present paper proposes the use of a multimethod approach to investigate queue development as it affects patients' access to a public adult ICU (ICU-A) in Rio de Janeiro and to propose policy strategies, considering that critical care network organization is a complex public health problem.

## METHODS

This study was approved by the *Universidade Federal do Rio de Janeiro* Ethics Committee (project number 11257513.3.0000.5257).

According to Mingers and Brocklesby,^(8)^ multimethodology is the "art" of using more than one methodology or parts of different methodologies in combination to consider the various aspects of real world problems. This is especially useful to capture peoples' viewpoints and values, which cannot be represented mathematically but must be evaluated by rigorous and systematic nonquantitative methods. Hence, the use of a combination of methods is necessary in order to tackle the various aspects of the problem; both quantitative and qualitative models are used to help understand the complex and "wicked" problem.^(9)^

Herein, the multimethodological approach considers two main axes: quantitative and qualitative.

### The first axis: quantitative estimation of public adult intensive care unit beds

The study investigated a retrospective cohort made up of adult patients requesting admission to a public ICU-A with 268 beds, regulated by the CER in Rio de Janeiro from 2010 to 2011 (Table 1).

**Table 1:** Intensive Care Unit beds regulated by the Regulatory Center (Central Estadual de Regulação - CER) between 2010 and 2011

Hospitals	Total
Hospital Estadual Adão Pereira Nunes	08
Hospital Estadual Albert Schweitzer	18
Hospital Estadual Alberto Torres	06
Hospital Estadual Azevedo Lima	08
Hospital Estadual Carlos Chagas	20
Hospital Estadual Getúlio Vargas	47
Hospital Estadual Prefeito João Batista Cáffaro	02
Hospital Estadual Rocha Faria	06
Hospital Estadual Roberto Chabo	16
Hospital Estadual Vereador Melchiades Calazans	10
Hospital Federal do Andaraí	16
Hospital Federal Cardoso Fontes	08
Hospital Federal de Bonsucesso	17
Hospital Federal de Ipanema	10
Hospital Federal da Lagoa	25
Hospital Federal dos Servidores do Estado	35
Total	268

Source: Departamento de Informática do SUS (DATASUS).

CNES - Cadastro Nacional dos Estabelecimentos de Saúde [Internet]. 2014 [cited 2018 Aug 28]. Available at: http://datasus.saude.gov.br/sistemas-e-aplicativos/cadastros-nacionais/cnes

The number of beds necessary to meet the demand was estimated by time series and queuing theory in four different scenarios. These scenarios considered the number of queue entries (by arrival rate and medical requests) and differing LOS in the ICU, shown in table 2.

**Table 2:** Parameters for each scenario

Scenarios	Arrival rate (patients/hour)	Length of stay (days)
I - Current demand and LOS	Total requests	Mean public (regulated) LOS
II - Reduced LOS	Total requests	Mean LOS (public and private ICU)
III - Reduced demand	(Total requests - queue abandonments)	Mean public (regulated) LOS
IV - Reduced demand and LOS	(Total requests - queue abandonments)	Mean LOS (public and private ICU)

LOS - length of stay; ICU - intensive care unit. Fonte: Departamento de Informática do SUS (DATASUS). Estabelecimentos com tipo de atendimento prestado - internação [Internet]. 2013 [cited 2018 Aug 28]. Available at: http://tabnet.datasus.gov.br/cgi/tabcgi.exe?cnes/cnv/atintbr.def

**Figure 2:**
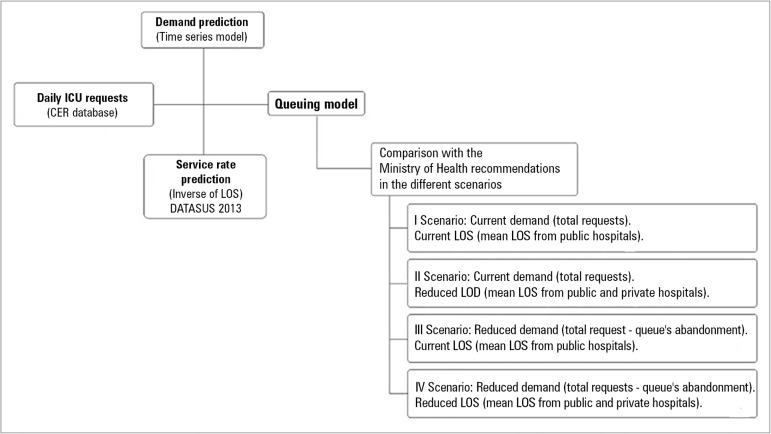
Methodological steps from demand prediction (based on time series) to the number of intensive care unit beds (based on queue theory) for the different scenarios.^(11)^ CER - *Central Estadual de Regulação*; LOS - length of stay.

The findings were compared with the Brazilian government's recommendation that the number of ICU beds be a proportion of the total population. In the case of Rio de Janeiro, the recommended range for adult public beds is 118 - 353. The methodology is shown in figure 2.

A single queue was considered, including all 268 adult ICU beds, and bed capacity was estimated by the minimum number needed to ensure system stability, assuming a maximum six-hour wait time before ICU admission. A six-hour wait time is considered clinically feasible to sustain an unchanged prognosis if there are appropriate prehospital and emergency support measures available.^(1,10)^ Finally, the queue was organized by clinical priority, according to the CER distribution model.

In queuing theory, the model is based on the interaction between two parameters: mean queue arrival rate (λ = patient/hour), representative of demand; and mean service rate (µ) or exit rate (1/µ = inverse of LOS), representative of bed supply.

Once the queue system entry (λ) and exit (µ) parameters have been defined, it is possible to determine for different scenarios the minimum number of beds required, maximum wait times and probability curves.

Detailed methodology can be seen in Goldwasser et al.^(11)^ and Angelo et al.^(12)^

### The second axis: qualitative analysis and focus group

The focus group (FG) is a qualitative methodologic strategy that can be used to understand the challenges regarding the process of ICU queue formation in clinical and management practice. It enables exploration of interviewee's perceptions and provides insight into clinical and management behavior in their own words.^(13)^ The participants are gathered in groups, and a moderator facilitates the evaluation of concepts, the identification of problems, and the clarification of points that are still obscure from the answers obtained in the quantitative analysis.^(14-16)^

To encourage openness in responses and to reduce potential bias and influence on participants, it was decided to run two separate FGs based on their specializations: 1 physicians working in the ICU and 2 physicians working in the Emergency Room, both from public hospitals. The FG sessions were conducted based on the methodological orientation and theory of content analysis.^(16)^ The interview technique was driven by a script. The 32-item consolidated criteria for reporting qualitative research (COREQ) were followed in this study.^(13)^ Sampling was used for data saturation to establish or shorten the length of the interview.^(17)^ The interviews were audio recorded and transcribed verbatim. No specific software program was utilized to manage data collected.

To preserve the contributors' identity and anonymity, alphanumeric codes were used in the transcript of their testimony. The Emergency Room group was identified as E_1_, E_2_, E_3_ ..., and the ICU doctors were identified as U_1_, U_2_, U_3_ ....

A sample size of 5 to 10 participants has been reported to be sufficient for FGs and to enable data saturation to be met.^(16,17)^ The characteristics of the FGs are demonstrated in table 3.

**Table 3:** Characteristics of the doctors from the Focus Group

Area	Sex	Age (years)	Graduating (years)	Specialist (years)	Specialty
ICU	F	43	20	6	Intensivist
	M	45	23	7	Intensivist
	M	40	15	8	Intensivist
	M	55	31	7	Intensivist
	F	51	18	8	Intensivist
Average		46.8	21.4	7.2	
Emergency room	M	35	13	8	Neurosurgeon
	F	30	7	6	Internal medicine
	F	48	24	22	Pediatrician
	F	44	20	22	General surgeon
	M	40	16	14	General surgeon
Average		39.4	16	14.4	

ICU - intensive care unit; F - female; M - male; ER - emergency room.

All of the participants were informed about the general purpose of the survey, their confidentiality rights and the recording of the interview, and a written Informed Consent was obtained.

The steps of content analysis are described below:^(14,15)^

1. A general reading of the transcribed material was completed, with the purpose of grouping the quotations (QU).
2. The vast amount of data was grouped by the frequency of which they appeared, by the emotional burden they were carrying, and the similarity, complementarity or differences of the QU. Then, the QU were grouped thematically to subthemes and themes. The themes emerged on their own; they were not derived from existing theories or previous related studies.
3. The results were interpreted and evaluated in order to reveal the manifest and latent content (inferences).

## RESULTS

### First axis: quantitative analysis

In 2010-2011, the State of Rio de Janeiro had 4,219 ICU-A beds. Of those, 1,007 were public beds (SUS), but only 268 were regulated by the CER. In the analyzed period, there were 33,101 medical requests for ICU-A admissions, and a waiting queue was formed. During that period, 25.0% of the individuals were admitted to the ICU, 55.0% abandoned the queue and 20.0% died before admission. Among the main causes for abandonment, 47.0% were related to clinical improvement or recovery, 35.0% accessed ICU beds by other means, and 9.0% were outside of the regulation profile.

Table 4 presents the results obtained in each scenario that was studied. To ensure a maximum wait time of six hours for 95.0% of the patients, it would be necessary to increase the number of ICU-A beds to 628 in scenario I (the current scenario), 366 in scenario II, 471 in scenario III and 275 in scenario IV. Only the number of beds calculated for scenario IV (254 - 275) is within the range recommended by the Brazilian Ministry of Health.

**Table 4:** Parameters and results obtained for each scenario

Scenarios	I	II	III	IV
Arrival rate (patients/hour)	2.19	2.19	1.63	1.63
Length of stay (days)	11.3	6.5	11.3	6.5
Service rate (patients/hour)	0.004	0.006	0.004	0.006
Number of ICU beds for a maximum 6 hour wait time	628	366	471	275

ICU - intensive care unit; Scenario I: current demand + current length of stay; scenario II: current demand + reduced length of stay; scenario III: reduced demand + current length of stay; scenario IV: reduced demand + reduced length of stay.

### Second axis: qualitative analysis

**Figure 3:**
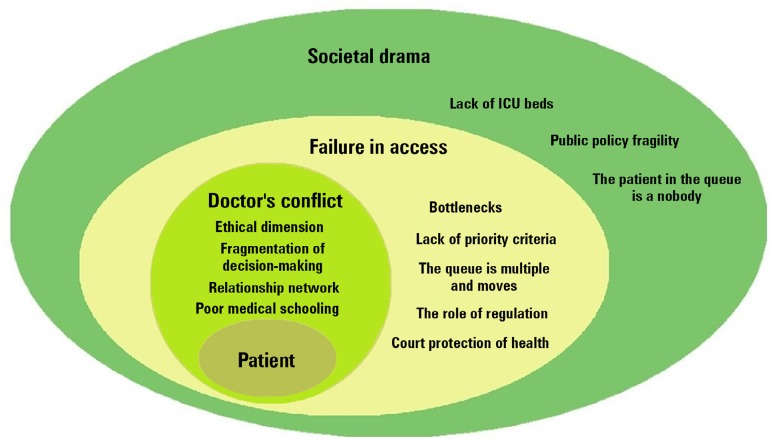
Hierarchical perceptions of a complex societal health problem. The doctor's dilemma expresses the individual dimension with 76 quotations. Ethical issues have been the most cited subtheme. Failure in access addresses equity and was the theme with the most citations (84). The big picture, or the societal drama, exposed the weakness of public policies, the lack of intensive care unit beds, and the lack of accountability as barriers for patients' access to the intensive care unit (48 quotations). ICU - intensive care unit.

Two hundred and eight (208) quotations (QU) were recorded from the FGs. They were grouped into 12 subthemes and 3 themes. As a result, the perceptions of the ER and ICU staff could be organized into 3 different hierarchical dimensions: individual (the doctor's conflict); collective (failures of access and equality); and broader societal factors (societal issues and citizenship). They are represented by the frequency of the QU in figure 3. Sample statements from the interviews are shown below and exemplified in table 5.

**Table 5:** Main comments from physicians in the focus groups

**The medical dilemma**
**U2:** … there is a patient, “Mr. X,” outside of the hospital with “this and that” and he needs the ICU. I have three “Mr. Xs” just in here at the ER. And my “Mr. X” is younger. My problem is here, not there.
**E4:** “He would benefit from the ICU, and I have already asked for his admission. But he will have to wait for ICU bed availability”. This is my speech to the family.
**E5:** “I lose the patient” because there is no ICU bed available.
**U2:** ...we are afraid to discharge the patient from the ICU. Here at the ICU, we have a multidisciplinary team that is better than the outside.
**E5:** The nurse usually asks us to choose. She says: “Doctor. There are 3 patients and just one bed available. Whom do you give priority?”
**U5:** I don’t care who comes to the ICU. I think the CER regulates the patients. They will decide. I must admit the patient, prescribe, treat him and discharge him quickly.
**U2:** The CER should have a kind of a “Superman telephone”. I don’t need the hospital director involved with ICU patients admissions. I need a CER with some empowerment to say: “I have a patient with a life-threating condition, and I need to take him to the ER. And then he would be visible to the ICU staff.
**E4:** I exchange messages by WhatsApp.
**U2:** The chief doctor from the ER says: “I have a decompensated patient with diabetes.” That’s how it works.
**The failure in access**
**U3:** Our worst enemy is an inadequate indication for the ICU. Suppose there is an elderly patient with a nonreversible neurologic disease. The ER colleague thinks that he needs ICU just because he is on mechanical ventilation.
**E4:** All patients with a higher risk of death have the right to be admitted to the ICU. I think that the criteria should be first come, first served.
**E5:** No. We don’t have protocols for ICU admission.
**U1:** Sometimes the patient leaves the ICU and returns very quickly. He has just arrived at the general ward and hasn’t even “warmed up” the bed when he develops a retained airway secretion, and nobody was able to help him.
**U2:** The queue is not unique. I also have the demands from the hospital, like the surgical patients... they compete.
**E1:** The problem is the number of nurses outside the ICU. They must give medicines, feed the patients, turn them every 2 hours. How can they offer good care?
**U3:** There are no oxygen therapy devices in the general ward. How can we discharge patients from the ICU?
**U4:** Usually, there are few nursing beds for admitting patients after ICU discharge. I think this is the most important reason for patients’ prolonged length of stay.
**E1:** A patient’s family asks for a medical report. It must be written that he is at risk of death and he needs the ICU. And the judge determines his admission to any available ICU.
**The societal drama**
**E1:** “The queue will explode”!
**E1:** (The queue) happens because there are no ICU beds available. It’s a shame. Only nine ICU beds in a hospital that is a referral source for the Olympic games!
**E4:** It is not only the lack of ICU beds. ...if you don’t invest in primary care and public safety. It is cheaper to treat hypertension and diabetes instead of cerebral vascular disease.
**E1:** He is a nobody, and I nominate him to the ICU to become somebody.
**E1:** ... every day we admit a patient who has been shot… every day. People crashing into violent traffic. An amazing number of motorcyclists. It won’t solve the problem creating more ICU beds if there is not a decline in what makes them need to come here.
**U4:** if we could bring this line to the hospital... we'd have to deal with only one part of the problem: Which one will benefit from the ICU? Patients must go to the ER, be first evaluated, and then go to surgery, or have a computed tomography scan or go to the ICU."

ICU - intensive care unit; ER - emergency room.

### Theme 1 - The medical conflict

This topic focuses on the individual dimension. Not surprisingly, concerns about ethics were the most frequently cited subtheme. Clinicians know that there are patients waiting in a queue outside of the hospital, despite the fact that this not visible to them. They are simultaneously in direct contact with patients inside of the hospital who are competing for the same available ICU beds. Doctors work under pressure from families, colleagues and the hospital manager to choose which patient would receive the greatest benefit from the ICU. Their concept of priority will vary according to their own judgment and interpretation of social responsibility, accountability and transparency.

In general, the FG did not consider the CER and its regulatory role to be legitimate, and the members assumed that the priority decision-making process was their own. In daily practice, personal contacts among health professionals, phone calls and *WhatsApp* create a nonofficial parallel network inside hospitals.

On the demand side, the FG perceived a need to train professionals working in prehospital care to identify critical patients and start first aid in a timely manner. In the opinion of the FGs, the prehospital staff is usually made up of young and unprepared doctors.

Regarding ICU bed management issues, although doctors are aware of the importance of reducing LOS, ICU physicians tend to delay discharging their patients because they believe care will be worse on the ward.

### Theme 2 - The failure of access

The rationale behind the failure of access addresses the issue of equity, and opinions about it varied among clinicians from the ER and ICU; it was the theme with the highest number of citations. The system advocates for universal care, but there is not enough room for everyone.

Differing thoughts about the priority criteria depend on the position that the physician holds within the system. There are conflicting opinions regarding what constitutes a patient's timely admission to the ICU. For ER physicians, the queue consists of every critically ill patient in the emergency room, and all of them need to be admitted in the order of their arrival, without regard to protocol. In contrast, ICU physicians prioritize clinical severity and recognize diverse simultaneous queues within the hospital, from other hospitals and from the regulatory center. In addition, judicial decisions skip ahead in the line without medical evidence of benefit, and this generates great frustration among doctors.

Topics regarding the lack of infrastructure in the general wards and an unprepared staff were raised by the doctors as barriers for both patients' admission and discharge, which impacts access. Additionally, a lack of accountability and motivation of some professionals creates huge difficulties for bed flow.

### Theme 3 - The societal drama

The societal dimension, or the "big picture", is relevant and was exposed during interviews. The weakness of public policies is strongly related to the high demand and low supply of public ICU beds. The participants stated that uncontrolled urban violence is a serious cause of the increased number of critical patients. They also pointed to the inadequate level of investment in primary healthcare and to the unsanitary conditions that expose the population to diseases that ultimately culminate in high ICU demand. The lack of citizens' education and better health habits are causes of the high prevalence of cardiovascular diseases and diabetes, for instance.

Another societal drama experienced by the physicians who were interviewed is that no one is accountable for the patients in the queue. The quote, "*He is a nobody, and I nominate him to the ICU to become somebody,*" summarizes the doctors' consensus that patients, either in the queue, the ER department, or the ICU, should be treated with a more humanitarian approach.

## DISCUSSION

Healthcare service planning is a complex societal problem requiring multidisciplinary and multidimensional approaches to organize the relationships among its components.^(18)^ In the case of the ICU's admission queue, arrival rates are influenced by epidemiological (an aging population), technical (medical evidence and technologies), cultural (curative instead of palliative care), ethical (priority criteria), organizational (regulations), and educational (prehospital care) issues. Service rates are driven by local guidelines but also by clinical decisions and conflicts.

The multimethodological design of this study brought together quantitative and qualitative research to deepen the understanding of the problem in Rio de Janeiro as a case study in order to optimize policies and joint solutions.

Although the current number of ICU-A beds regulated by the CER can be set within the population-based range that is advocated by the Ministry of Health by controlling arrival (input) and service (output) rates, the system remained unstable and did not guarantee opportune admissions for all critically ill patients during the analyzed period. There was an average of 2.2 new requests per hour for the CER, and if current input and output parameters were maintained, it would be necessary a to increase the number of ICU-A beds by 134% to guarantee the stability of the system with a 6-hour maximum wait time.

Otherwise, adjusting the number of ICU beds cannot be considered to be a unique solution for the problem, as shown by the network of relationships in figure 1. To complete the evaluation of the problem and look for solutions, the experiences and perceptions of the stakeholders, either by literature or by focus groups, are of utmost importance.

The analyzed situation generated a long queue with subsequent refusals of ICU bed requests, culminating in 20% of patients dying and 55% of patients abandoning the queue. The low entrance rate to the ICU of only 25% of requests observed in quantitative analysis underrepresented the absorption capacity of the UCI-A because the CER was competing for beds with other nonofficial (thus, invisible) queues created by physician networks inside and outside the hospitals. Additionally, some patients eventually skipped the queue by utilizing a judicial court order, which was only depicted in the qualitative FG analysis.

According to Metcalfe et al.,^(19)^ the avoidable mortality rate in patients who are refused admission to the ICU is high, particularly in emergency cases. Consequently, it would be reasonable to think that organizing the queue would reduce avoidable deaths. The strategy of *first-come-first-served*, by order of arrival, seems to be the most equitable, but would require a single line and enough ICU beds for everyone who needed one. Hence, even if each ICU offered the entirety of its beds for CER regulation, they would promptly be busier as a result of the hospital's internal demand. Only if those patients were truly regulated, by clear priority and ethical criteria, would the number of beds needed to maintain system stability be reduced.

Cochran and Roche^(20)^ observed that the prolonged queue waiting time in the ER induced patients to leave the hospital before receiving treatment. This was considered to be a serious public health problem and gave rise to a unit of measurement called *Door-to-Doc* or D2D, as an indicator of crowded service. In the quantitative study, it was noteworthy that a significant percentage of patients abandoned the queue due to clinical improvement, which was partially approached in the FG qualitative axis. First, one possible explanation would be that the prehospital care had been effective; second, it could be the result of an inappropriate *a priori* indication criteria. Adequate care in prehospital environments and following protocols might foster clinical and diagnostic improvements, thus avoiding erroneous indications for ICU admissions.^(1,10)^ According to the FGs, the unpreparedness of doctors to identify critical patients seemed to be the prevailing explanation. Additionally, the lack of legitimization of the CER criteria shows the fragility of the main policy to regulate and tackle the ICU bed shortage issue. The success of the Regulatory Centers, which are meant to be leaders in the process, relies on collaboration from all professional involved, and their ability to leverage change relies on establishing a dialogue with hospitals (both ER and ICU representatives) in order to guarantee better access and to implement clinical governance (CG) and network management (NM).^(21)^

To reduce the arrival rate (scenario III), other strategies need to be implemented, such as strengthening and legitimizing regulatory guidelines and policies (from the CER) to select the patients who will most benefit from intensive care.^(22)^ Improving communication^(23)^ and shared decision-making also contributes to rationalizing the available resources. To avoid unnecessary admissions, it is important to revisit the concepts of palliative care and futile treatment, and whenever necessary, accept the recommendations from the Ethics Committees.^(24)^

Scenario II was a model for improving the management of patient flow by reducing patients' length of stay (LOS). When the average LOS was reduced by 58.0%, the need for new ICU beds fell by approximately 40.0%. Some of the reasons behind the longer LOS encountered in public ICUs were exposed in the FG, such as the fear of discharging patients to the unprepared and inefficient nursing ward. On the other hand, there was consensus about the importance of guidelines that influence internal processes related to prolonged stays in the ICU, such as excessive sedation,^(25)^ prolonged weaning from mechanical ventilation,^(26)^ and lack of specific measures for infection control.^(27)^ In terms of infrastructure issues, the FG recommended solutions like providing step-down units,^(28)^ resizing and reengineering the general ward, and creating palliative care units to decrease the average LOS. Finally, in terms of managerial and indicator analysis, it is important to associate LOS with severity-adjusted mortality rates, since a short length of stay may also be associated with the death of the patient.^(29)^ Studies have shown that the LOS tends to be longer in public than in private hospitals, despite similar mortality indicators, which might be associated with a selection bias.^(30)^

Finally, it is noteworthy that any policy dealing with critically ill patients is related to other health policies; it is interconnected with social issues such as community violence, and it affects the social rights of every citizen. The ER doctors' voices echoed public policies' fragility and the need to enhance primary care and to train doctors and nurses for prehospital and emergency care. Another alarming situation is that Brazil has one of the highest homicide rates in the world, 71.9% of which are committed by firearms, contributing to demand for the ICU.^(31)^ Soranz et al.^(32)^ draws attention to the low availability of primary care health teams in Rio de Janeiro, the lowest among the Brazil's capital cities, which was only 40% in 2013.

A limitation of this study is that only regulated ICU beds were considered in the analysis. Moreover, no distinction was made between major groups of diseases, such as postoperative, cardiovascular, neurosurgical, and trauma, which might have different impacts on queue entry and exit. Ideally, the model would be able to consider multiple waiting lists. Another limitation, concerning focus group analysis, was the lack of other social representation, such as nurses, physicians from clinical wards and from regulatory centers.

## CONCLUSION

Queue formation should be treated as a complex societal problem with a multifactorial origin, interrelated solutions and strong social impact.

There is a shortage of public adult intensive care unit beds in Rio de Janeiro, and the equation to solve the problem cannot be reduced to arrival and service rates since there are many ethical, epidemiological, cultural and organizational issues that influence intensive care units capacities. Organizing the queue through priority criteria - since there are not enough beds for everyone - and implementing protocols to reduce length of stay are important measures, but their applications are nested in a network of relationships that must be taken into consideration.

In the short term, it is essential to redefine and consolidate the regulatory center as a fundamental policy to manage the demands of critical patients and provide available resources in a timely manner. In this sense, the *Central Estadual de Regulação* may work together with all stakeholders to guarantee clinical governance and consolidate network organization.
